# Orphan diseases of the nose and paranasal sinuses: Pathogenesis – clinic – therapy

**DOI:** 10.3205/cto000119

**Published:** 2015-12-22

**Authors:** Martin Laudien

**Affiliations:** 1 Department of Otorhinolaryngology, Head & Neck Surgery, University Medicine of Kiel, Christian-Albrechts-University, Kiel, Germany

**Keywords:** nose, paranasal sinuses, orphan disease, rare disease

## Abstract

Rare rhinological diseases are a diagnostic challenge. Sometimes it takes months or even years from the primary manifestation of the disease until the definitive diagnosis is establibshed. During these times the disease proceeds in an uncontrolled or insufficiently treated way. (Irreversible) damage results and sometimes life-threatening situations occur. The unexpected course of a (misdiagnosed) disease should lead to further diagnostic reflections and steps in order to detect also rare diseases as early as possible.

The present paper discusses granulomatous diseases of the nose and paranasal sinuses caused by mycobacteria, treponema, Klebsiella, fungi, and protozoa as well as vasculitis, sarcoidosis, rosacea, cocaine-induced midline destruction, nasal extranodal NK/T cell lymphoma, and cholesterol granuloma. Furthermore, diseases with disorders of the mucociliary clearance such as primary ciliary dyskinesia and cystic fibrosis are presented, taking into consideration the current literature.

## 1 Introduction

Beside frequent sinonasal diseases such as viral rhinosinusitis, rare rhinological diseases dealt with in this paper play a subordinate role from a socio-economic point of view. However, the definitive diagnosis for patients is sometimes preceded by years of suffering and uncertainty. Mostly irreversible damage has occurred before an adequate therapy is initiated. Besides its central position in the face and the resulting importance for social interaction, the nose has several important functions. The nasal mucosa has the function of acclimatizing the breathed air, it is the first organ of innate and acquired immune defense, and it represents a physical barrier (mucus, ciliary function). Afferents for reflexes (e.g. coughing, sneezing) as well as olfaction are further important components of the rhinological system. Rare diseases of this system can quickly lead to a lethal threat for patients as they are mostly very difficult to diagnose because the symptoms and findings often overlap [1], [2], [3], [4].

### 1.1 Definition of orphan disease

Worldwide, the definition of orphan diseases is inconsistent, basing on a prevalence of 1–8/10,000 [5]. In Europe, a prevalence of not more than 5 patients on 10,000 inhabitants is considered as rare disease [6]. Diseases are classified as orphan regarding the geographical and historical as well as age-related context. Rhinoscleromatosis is a frequently occurring infectious disease in some parts of India involving the nasal cavity. In Europe, this disease is rare and appears mostly in patients coming from endemic areas [7], [8]. In contrast, in Germany tuberculosis is still not classified as rare disease with an incidence of 5.2 per 100,000 inhabitants in 2012 [9], however, the nasal involvement is rare (less than 1 of 476 patients [10]). The involvement of the nose in cases of sarcoidosis is rare (1–4% of all patients). Regarding the worldwide incidence of 18/100,000 people, however, this disease is not defined as orphan. Considering the incidence related to the age, sarcoidosis is rare in patients younger than 15 years (1/100,000) and even very rare in children younger than 4 years (0.06/100,000) [11]. 

In the following overview, granulomatous diseases of the nose and the paranasal sinuses as well as special diseases with disorders of the mucociliary clearance of the nasal mucosa are discussed on the basis of the current literature.

## 2 Granulomatous diseases of the nose and paranasal sinuses

Histological criteria for granulomas are not defined in a uniform way. Mostly, granuloma is described as an inflammatory nodular tissue reaction with involvement of epitheloid cells, mononuclear cells, and giant cells. The reasons for the development of granulomas are various. They are summarized in Table 1 (Tab. 1).

### 2.1 Mycobacteria

#### 2.1.1 Tuberculosis (TB)

Tuberculosis is a chronic bacterial infection mainly caused by* Mycobacterium (M.) tuberculosis*. The disease is characterized by the development of granulomas in the infected tissue and cell-mediated hypersensitivity.

Nasal infection is rare (4 of 1,486 patients with proven tuberculosis). Primary nasal infection is very rare (12 of 35 patients with nasal tuberculosis), often there is a secondary (hematogenous or lymphatic) infection after pulmonary manifestation. Primarily, nasal infection is located at the nasal septum (in 87% of the cases) and lateral nasal wall (inferior turbinate in 12.5% of the patients), predominantly unilateral. The competent nasal mucosa harbors sufficient defense mechanisms (mechanical and bactericidal effect of nasal secretion) against *M. tuberculosis*. Immune compromised patients (HIV infection, malnutrition), women, and middle-aged people are particularly affected.

Nasal obstruction, rhinorrhea, foul-smelling crusts, epistaxis, and painless or painful lesions up to septal perforation are unspecific local symptoms. B symptoms may occur. At first, only thickened nasal mucosa appears followed by red or pink, easily bleeding granulomas or ulcerations. Secretion is usually very thin. Soft tissue connections, for example the lacrimal drainage system, allow an involvement of the outer skin by continuity. Furthermore, preferably the nasal skin (90% of the patients show involvement of the skin of the head and neck region) might be involved due to lymphatic or hematogenous spread. This involvement usually appears as lupus vulgaris with destruction.

Histological examination of nasal biopsies is usually sufficient and is completed by microbiological, serological (Quantiferon®-TB) and radiographic examinations, as well as skin tests (Mantoux test).

The standard treatment is a multiple antibiotic therapy (isoniazide, rifampicine, pyrazinamide, streptomycin/ethambutol) for several months. Currently, increasingly (multi-)resistant strains are detected so that therapy with so-called reserve antibiotics has to be administered. Involvement of the paranasal sinuses may also require surgical therapy. After healing, reconstructive surgery is performed, if necessary [1], [10], [12], [13], [14], [15].

#### 2.1.2 Leprosy, Hansen’s disease 

**Figure 1:**
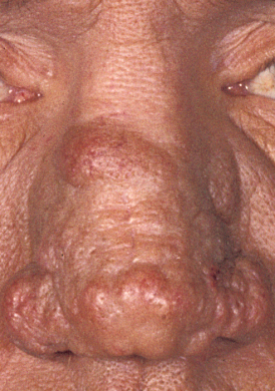
Tuberculoid leprosy

Leprosy (Figure 1 (Fig. 1)) is a rare chronic infectious disease caused by the obligate intracellular *Mycobacterium (M.) leprae* (especially in macrophages and Schwann cells). The pathogen cannot be cultured but grown in mice paws and armadillos. The optimal growth temperature is 27–30°C. This fact may contribute to the high nasal load.* M. leprae* is little infectious and pathogenic and shows an incubation time of up to several years. Despite worldwide efforts to eliminate the disease, the number of newly diagnosed cases worldwide (in 2012) was 232,847 (incidence: 0.4). In Germany, 5 cases were registered in 2012.

Up to now, the transmission pathways are not fully understood. The nasal mucosa seems to play an important role in the transmission of the disease. The ultimate reservoir of infection seem to be humans. Carrier with subclinical course may be a cause of transmission. Treatment of germ carriers could lead to further success in the fight against this infectious disease. 

Leprosy is classified in four types:

- Lepromatous leprosy
- Borderline leprosy
- Tuberculoid leprosy

And their transitional forms, and

- Subclinical leprosy

Clinically, the disease is characterized by the infestation of the skin and peripheral nerves. The nasal mucosa, however, is predominantly involved, though not clinically leading (in 90% positive detection of pathogens in the nasal mucosa). Based on this observation, diagnostic efforts should focus on the nasal mucosa. 

After exact analysis, changes of the nasal mucosa can be found in a high proportion of patients (more than 90%). Specifically those are initially:

- Rhinorrhea
- Pale, yellowish thickened mucosa
- Nodular infiltrates
- Flat plaques or nodules up to 5 mm

In the further course of the disease:

- Nasal obstruction
- Ulceration
- Crusts
- Sensory disturbances
- Hyposmia
- Destruction of cartilage and bone (with deformity of the external nose and chronic atrophic rhinitis)
- Epistaxis

Genetic and immunological properties of the individual patient seem to significantly affect the course of the disease. The tuberculoid form is frequently observed in patients with a high cell-mediated immune response of the Th1 type. The course of the lepromatous form is associated with a low cell-mediated immune response of the Th2 type. Based on the definition of the individual’s immune status, the course of the disease can be predicted which should be included in the therapeutic decision. The special nasal diagnostics consist of careful examination of nasal swabs/brush biopsies, nasal secretion, and nasal biopsies applying microscopic and molecular methods.

The treatment includes several months of multiple antibiotic therapy with rifampicin, dapsone, clofazimine, minocycline, ofloxacine, or clarithromycin and possibly surgical reconstruction [9], [16], [17], [18], [19], [20], [21], [22], [23], [24], [25], [26], [27], [28].

### 2.2 Treponema

#### 2.2.1 Syphilis 

**Figure 2:**
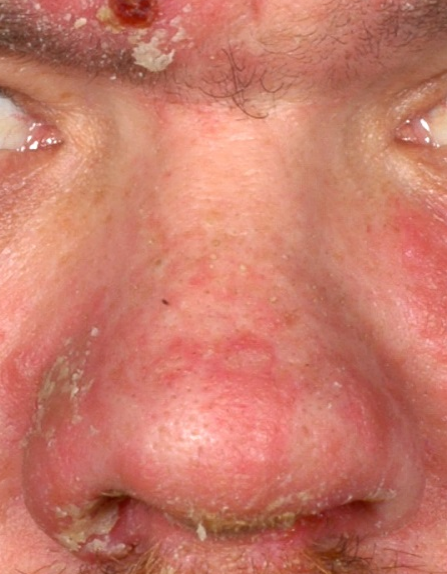
Syphilis, tertiary stage

Syphilis (Figure 2 (Fig. 2)) is an infectious disease caused by the spirochete *Treponema pallidum*. The disease is not a rare disease by definition (incidence in Germany in 2012: 5.4/100,000, since the 1990s: again rising incidence); nasal involvement, however, is very rare. The causative agent is pathogenic only for humans and it is transmitted through sexual contact, placenta, or in the birth canal.

For therapeutic reasons, the disease is classified in an early (primary and secondary syphilis within one year after infection) and late syphilis. Progression of the primary lesion is detected only in part of the infected patients.

Nasal involvement is mainly found in late syphilis in the tertiary stage. The mucinous defense and the ciliary function of the nasal barrier are disturbed. Hyperergic response to pathogens leads to the granulomatous reaction of the tissue with soft circumscribed swelling and inflammatory mucosa with ulceration. The septum is the preferred site followed by septal perforation and saddle nose deformity, but also the lateral wall can be affected. If left untreated, nasal involvement leads to stenosis and atresia. A nasal chancre (ulcus durum, hard chancre) is rare and localized at the vestibule of the nose. The equally rare secondary stage appears as unspecific rhinitis.

Besides, syphilis of newborns (congenital syphilis) is divided into an early, late, and stigmatizing phase. India shows an incidence of about 1% of newborn syphilis and the amount is rising also in Europe (primarily due to immigration). In the early stages, there may be infectious rhinitis and thickening of the mucous membranes (including bullous mucosa, occurring in the 1^st^–5^th^ month of life) and in the stigmatizing phase saddle nose deformity may occur due to defects of the nasal support structures.

The course of the disease is rapid and severe and frequently coincides with HIV infection.

The diagnosis is made based on clinical, histopathological, serological, and microscopic analysis.

Antibiotic therapy is applied with penicillin, and in cases of penicillin intolerance, cephalosporins, macrolides, or tetracyclines are administered for several weeks [29], [30], [31], [32], [33], [34], [35], [36], [37], [38].

### 2.3 Klebsiella

#### 2.3.1 (Rhino-)scleromatosis

In Germany, rhinoscleromatosis is a rare disease caused by the predominantly intracellularly occurring gram-negative bacterium *Klebsiella (K.) pneumonia ssp. rhinoscleromatis*. In Africa, Southeast Asia, Mexico, Central and South America, and parts of Eastern Europe, the disease is still endemic. The transmission of pathogens occurs probably airborne. Overpopulation, malnutrition, and poor hygiene standard seem to increase the likelihood of infection. Disease cases outside endemic areas are usually based on migration of the patients.

Typically, the disease proceeds in three stages. The initial catarrhal stage is followed by the proliferative stage leading to the fibrotic stage. In almost 100% of the cases, the nasal mucosa is affected, mostly bilaterally (75%). Following the common-airway concept in descending order of frequency, the pharynx (18–40%), the larynx (5%), the trachea (30%), and bronchi (2–7%) are affected. In rare cases, there may be a manifestation of the skin, the nasolacrimal duct, or the premaxilla. In most cases, the lymphatic system is not affected. Women are equally affected as men. The preferred age seems to be 3^rd^ and 4^th^ decade of life.

A shift observed in the ratio of CD4/CD8 positive lymphocytes could lead to disruption of the defense against infection in affected patients or it represents an epiphenomenon. Likewise, a genetic pattern (nicotinamide adenine dinucleotide phosphate oxidase complex, HLA-DQA1*0311-DQB*0301 haplotype) may contribute to disease exacerbation. Evidence of a positive family history could speak for human transmission or a genetic predisposition.

In the catarrhal stage, the patients complain about foul smelling purulent rhinorrhea, nasal obstruction, and crusting. Atrophy of the nasal mucosa is observed endoscopically. In the following stage, the patients complain about epistaxis, nasal deformities, anosmia, epiphora, and partial anesthesia of the soft palate. Endoscopically blue-reddish granulomatous lesions are detected. In the fibrotic stage, increasing deformities of the nose appear, with collapse of the supporting structures, sometimes also hoarseness and stenosis of the aerodigestive tract. 

The diagnosis is made with reference to the microbiological detection of bacteria (in 50–70% of the first biopsy in the proliferative stage and in about 60% of cases in the fibrotic stage), and by histological examination of biopsies from affected areas. Plasma cells, large foam cells (so-called Mikulicz cells), where bacteria are detected, and Russell bodies (probably converted plasma cells) can be detected. The microbiological differentiation is difficult and can only be performed in specialized laboratories.

Imaging reveals the extent of the disease. Especially in the proliferative stage, homogeneous well-defined tumors of the nasal cavity can be detected. The turbinates are regularly involved and frequently the nasal septum shows destruction. A growth into adjacent structures (sinus, orbita, skull base) is possible.

Treatment is primarily performed by systemic antibiotic therapy for several weeks or months, sometimes up to the lack of detection of bacteria and the typical histological findings in serial biopsies, with gyrase inhibitors (fluoroquinolones, such as ciprofloxacin), or tetracyclines (doxycycline). Surgical therapy is reserved to acute infection status in case of complications. Especially the aerodigestive tract may be compromised by granulomatous tissue with the need of vaporization with laser or electrocoagulation. After healing, reconstructive surgery of functional and/or esthetic reasons may be necessary. However, it should be noted that recurrences are frequent (up to 25% in 10 years) [7], [39], [40], [41], [42], [43], [44].

### 2.4 Mycosis 

#### 2.4.1 Histoplasmosis (reticulo-endothelial cytomycosis) 

Histoplasmosis is a highly infectious systemic mycosis caused by the dimorphic fungus *Histoplasma capsulatum*. The fungus is endemic in North, Central, and South America. After inhalation of fungal spores, they are phagocytosed by macrophages in the lungs and transform into its yeast form. The incubation period amounts to some days to several months. A peak incidence occurs around the 3^rd^ to 4^th^ decade of life. For the development of the disease, the pathogen amount, the virulence of the fungus, and the immunological status of the patient are crucial predispositions. In the majority of cases, the infection is self-limiting or limited to the lung and usually asymptomatic. An occupational exposure is common (miners, cavers, farmers, archeologists). An involvement of the mucous membranes is extremely rare and usually due to an immuno-incompetence of the patient.

The nasal mucosa is erythematous, inflamed, and shows irregular ulcerations with crust formation. The inflammation is chronic (progressive), painful, and can progress to the outer skin. Nasal obstruction is an unspecific parameter. Sinusitis with cephalgia, nasal obstruction, and rhinorrhea is rare.

The diagnosis is made based on histopathological, microbiological, and molecular biological work-up of sample biopsies and immunological methods (e.g. histoplasma skin test). It takes 2–4 weeks to culture *Histoplasma capsulatum* so that therapy must be started immediately with a positive laboratory testing as a dissemination of the infection can be fatal. The direct evidence of pathogens by microscopy or molecular biological detection of fungal DNA from clinical material is available in specialized laboratories (e.g. consulting laboratory for imported systemic mycosis).

Nasal histoplasmosis is treated for several weeks to years with systemic antifungal therapy (azoles, amphotericin B). A therapeutic intervention with trimethoprin-sulfamethoxazole in less severe cases seems to be possible [37], [45], [46], [47], [48], [49], [50].

### 2.5 Protozoa

#### 2.5.1 Leishmaniasis

Leishmaniasis is caused by obligate intracellular protozoa of the genus Leishmania and depending on the species it is classified into a visceral, cutaneous, and mucocutaneous form. The mucocutaneous type is mainly caused by pathogens of the *Leishmania braziliensis* complex and transmitted by sandflies. Leishmaniasis occurs endemic in some parts of Europe (Turkey, Mediterranean areas). Increasingly, autochthonous cases are also reported in Germany. The proliferation of vectors and possible animal pathogen reservoirs may be an explanation for this observation.

The mucocutaneous form of the disease is usually severe with fatal courses. It can occur with a latency of several years after exposure. Primarily a manifestation of the skin and usually secondarily (in 94% of the patients) the (nasal) mucosa is affected. However, courses with synchronous occurrence of manifestations have been documented (2.7% of the patients with cutaneous leishmaniasis). Predominantly, male patients are affected. A genetically determined susceptibility, malnutrition, and impaired cellular immunity are described as risk factors for infection. In particular the course of the mucosal form of the disease may be affected by the infection of the protozoa by RNA viruses.

The manifestation at the inner nose shows edematous swelling, nodules, granulomatous lesions, redness, ulceration, rhinorrhea (about 50% of the patients), epistaxis (up to 65% of the patients), nasal obstruction (up to 90% of the patients), cephalgia, destruction of cartilaginous structures, and necrosis. In imaging studies, especially swelling of the nasal mucosa (96% of the patients) and destruction of the cartilaginous and bony structures (65% of the patients) can be detected. Patients with recurrent disease show a statistically significantly higher Lund-Mackay score. The problem of infection of the mucous membrane of the sinuses or secondary involvement of the sinus is unclear. Lessa et al. suggest a 5-step staging of nasal involvement, which is supposed to document the progress of the disease.

The diagnosis of the disease consists of immunological tests (Montenegro skin test), serological, histopathological, and immunohistological as well as cultural and molecular biological examinations.

Therapy depends on the causative species and consists of systemic administration of antimony, pentoxifylline, amphotericin B, or miltefosine. Not all courses of the disease require chemotherapy [51], [52], [53], [54], [55], [56], [57], [58], [59].

#### 2.5.2 Rhinosporidiosis

Rhinosporidiosis is a chronic granulomatous disease caused by *Rhinosporidium seeberi*. Currently, the organism is considered as being a eukaryotic parasite that does not belong to the fungi, and is subsumed in a new strain (Mesomycetozoea) with parasitic and saprophytic microorganisms that cause similar diseases in fish and amphibians. However, there are still groups that classify this microorganism as a fungus or prokaryote. *Rhinosporidium seeberi* is not cultured ex vivo. Rhinosporidiosis was first described by Seeber in 1900 and is endemic in Sri Lanka, parts of India, and Bangladesh. The ecological niche of the organism is unknown. An aqueous environment is assumed from which the germ penetrates the patient by micro-traumata. However, the disease also occurs in dry areas of the Middle East, so that there may be an environment-adapted form of the microorganism. Cellular and humoral defense mechanisms could be detected in humans and suggest the possible immunization of clinically healthy patients. Men are affected 2–4 times more frequently than females. The peak incidence appears between the first and third decade of life.

Rhinosporidiosis manifests predominantly on the nasal mucosa (70–85% of the patients). Less commonly, the nasopharynx, other mucous membranes of the upper aerodigestive tract, the skin, and internal organs are affected. Dissemination of the disease is rare and mostly fatal.

The nasal manifestation is characterized by vascular papillomatous or polypoid mucosal changes that can easily be damaged. The polyps are often stalked and unilateral. It is of particular note that the polyps “atypically” arise from the floor of the nose, the nasal septum, the inferior turbinate, the nostrils, and the nasopharynx.

Clinically non-specific symptoms of chronic rhinosinusitis with nasal obstruction, epistaxis, rhinorrhea, and bacterial superinfection are observed.

The diagnosis is made based on the histopathological detection of the pathogen.

The treatment of rhinosporidiosis consists primarily of surgery. The lesions are removed and cauterized basally. In cases of recurrences, steroid, dapsone, and amphotericin B are applied [60], [61], [62], [63], [64], [65], [66], [67].

### 2.6 Vasculitis

Granulomatous vasculitis belongs to the anti-neutrophil cytoplasmic antibody (ANCA)-associated primary small vessel vasculitides. Granulomatosis with polyangiitis (GPA, formerly Wegener’s granulomatosis) and eosinophilic granulomatosis with polyangiitis (EGPA, formerly Churg-Strauss syndrome) histologically show granulomas, vasculitis, and necrosis.

#### 2.6.1 Granulomatosis with polyangiitis (GPA, formerly called Wegener’s granulomatosis) 

**Figure 3:**
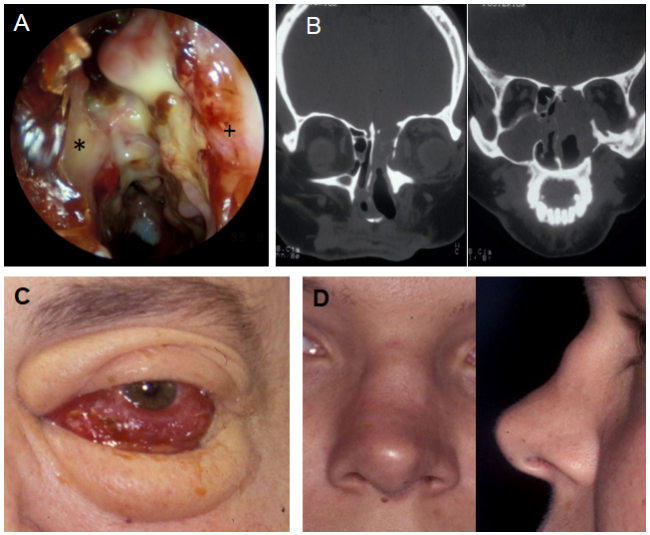
A) Nasal cavity in GPA with granulations, destruction, bacterial infection, left nasal cavity (choanal #, nasal septum*, lateral nasal wall+), B) CT scan of the same patient, destruction of the orbital wall and frontal skull base, C) The same patient, clinical aspect, D) Saddle nose deformity in GPA

GPA (Figure 3 (Fig. 3)) is a rare autoimmune systemic disease of unknown origin. During the course of the disease, in some patients fluctuating cytoplasmic (C-) ANCA with predominant proteinase (PR)-3 specificity could be detected. The ANCA titer does not predict the disease process. The disease can affect all organs of the body and in the 1930ies it was lethal within 4–7 months (first described by Klinger and Wegener). Today, the life expectancy is comparable to the general population due to modern immuno-modulating therapy.

An involvement of the head and neck region is diagnosed in about 80% of the patients in the course of the disease. In addition to the sometimes life-threatening subglottic manifestation, mainly a manifestation of the mucous membranes of the nose and paranasal sinuses is observed. 5% of the patients experience solely a so-called localized manifestation mostly with involvement of the nasal mucosa.

The symptoms of nasal involvement are unspecific with epistaxis, obstruction, crusting, olfactory impairment, rare cephalgia, and rhinorrhea.

The findings range from localized mucosal irregularity to granulations, ulcerations, bleeding, necrosis of the cartilage, bone, and mucosa as well as crossing the anatomical borders of the orbita and the skull base. The tissue destruction does not seem to be a sequel of changes of the blood supply but rather caused by (myo-)fibroblasts. Studies in animal models demonstrate the invasion of fibroblasts into cartilage grafts also at the opposite side of the mucosal (co-)graft so that migration and possible so-called homing factors are likely. The importance of the disturbed nasal microbiome and the disturbed nasal barrier is not fully elucidated for the initiation and progression of the disease. For certain bacteria (especially *Staphylococcus aureus*) increased rates of nasal colonization were found, associated with increased disease activity. The nasal barrier is disturbed both physically (decreased ciliary beat frequency) and chemically (antimicrobial peptides, pathogen associated molecular pattern (PAMP) receptors, and chemokines). The microbiome modulating therapy regime, especially with trimethoprim sulfamethoxazole seems to have a positive impact on the progression of localized disease forms.

The gold standard for diagnosis is the histology of the lesions. All histological criteria required are present only in a small percentage of biopsies (<20%). Close collaboration with pathologists is of crucial importance. Disease progression, further systemic manifestations, and serological investigations in an interdisciplinary framework, preferably in centers, quickly lead to the correct diagnosis. However, fulminant courses with permanent damage or rarely even death are possible today caused by the ambiguity of the symptoms and findings.

A systemic immuno-modulatory therapy is performed for several months to years with cortisone, methotrexate, cyclophosphamide, azathioprine, leflunomide, mycophenolate mofetil, etanercept, cyclosporine, and so-called biologicals (e.g. rituximab), primarily in combination with several drugs. Local mucosal treatment with saline solution rinsing, nasal ointments, and possible regular cleanings performed by an otolaryngologist leads to a relief of the symptoms. In primary diagnosis and during the course of the disease, a standardized examination and documentation seems to be necessary. In order to achieve this goal, Garske et al. proposed the Ear, Nose, and Throat Activity Score (ENTAS) [68]. Surgical interventions should be reserved to the treatment of complications in acute stages of the disease. In remission (at least 6 months), reconstructive surgery should be performed in cases of destruction (e.g. saddle nose, but also nasofacial, nasoorbital, and nasooral fistulas). Especially scarring and an increased susceptibility to infection even under low-dose immuno-modulatory therapy must be particularly considered [28], [68], [69], [70], [71], [72], [73], [74], [75], [76], [77], [78], [79], [80], [81], [82].

#### 2.6.2 Eosinophilic granulomatosis with polyangiitis

**Figure 4:**
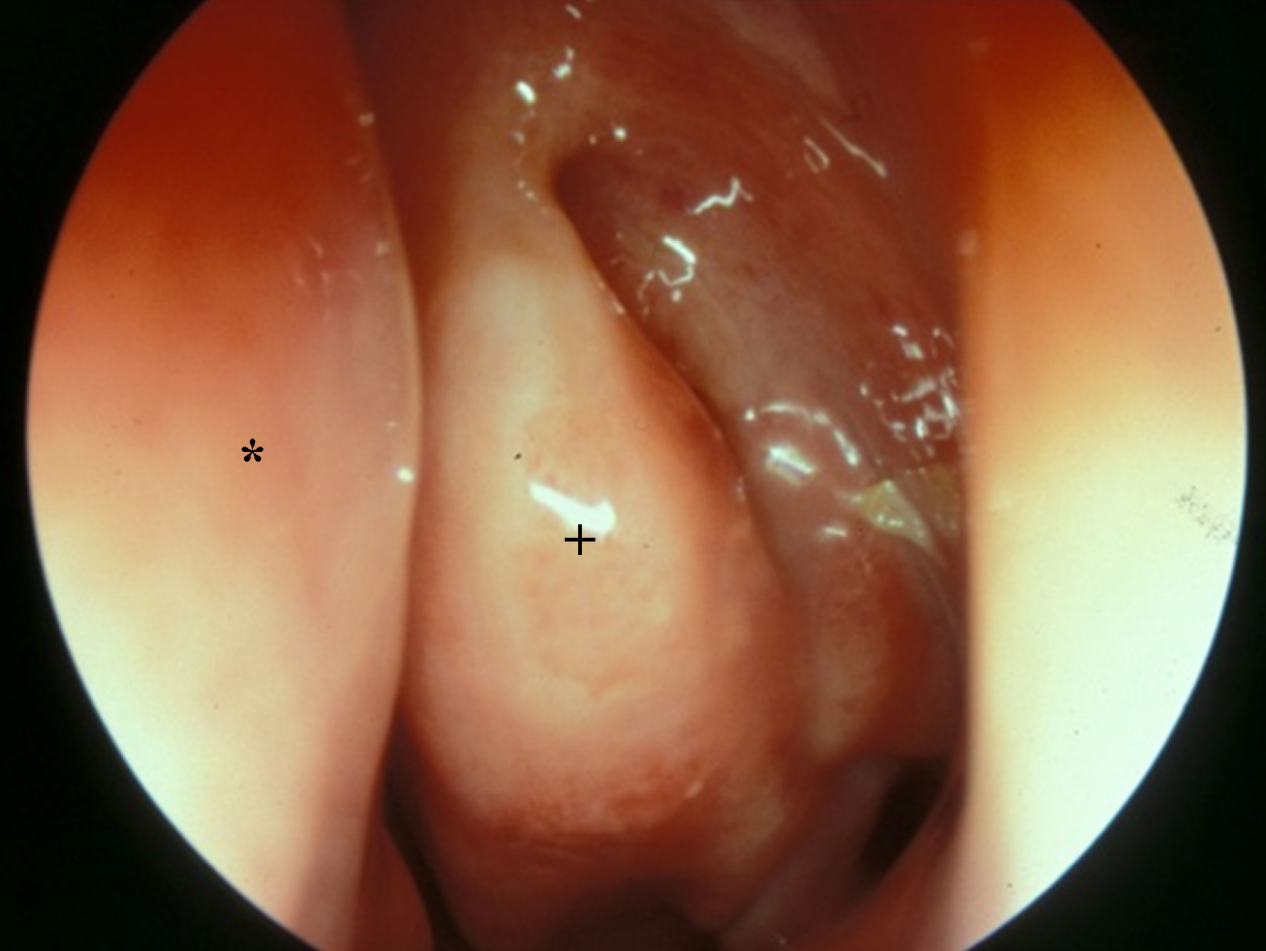
Involvement of the nasal mucosa in EGPA, middle meatus, left side (nasal septum*, medial nasal concha+)

Eosinophilic granulomatosis with polyangiitis (EGPA, formerly Churg-Strauss syndrome, Figure 4 (Fig. 4)) is a rare systemic autoimmune disease of unknown etiology. Myeloperoxidase (MPO) specific perinuclear (P) ANCA can be detected in a subset of patients, but the titer insufficiently represents the course of the disease. Interleukin 5 secreted by T cells under B cell influence appears to play an important role, especially for the survival of eosinophils in EGPA. EGPA can affect all organs. Especially the involvement of the heart and lungs can lead to life-threatening courses.

According to Lanham, the disease develops in three phases. In the prodromal phase, the patients suffer from asthma, allergic rhinitis, recurrent rhinosinusitis, and nasal polyposis [83]. The disease might persist in this stage for years. The following phase is characterized by peripheral blood and organ eosinophilia. This phase might also persist for years. In the third phase, organ- and life-threatening vasculitis occurs.

The nasal mucosa is thus early and regularly involved in the disease process. Scoring systems such as the Birmingham Vasculitis Activity Index (BVAS) and others based on experts’ agreement include an assessment of the head and neck region. Amazingly, in the current literature – with one exception – only single case reports and small case series analyzing the manifestation in the head and neck area are available.

Petersen et al. described an involvement of the head and neck region in 80% of 95 consecutive patients who were examined in a standardized multidisciplinary investigation from 1990–2010. 

In 42% of the cases, the patients complained of nasal obstruction, in 37% of rhinorrhea, in 21% of cephalgia, in 19% of crusting, in 16% and 13% of dysosmia and epistaxis, respectively.

Rhinoscopy revealed polyps in 43% of the patients, nasal secretions in 34%, hyperplasia of the inferior turbinates in 20%, synechia in 12%, and crust formation in 8%. In 40% of the patients, changes in the nasal mucous membranes like edematous swelling of vulnerable or non-specific mucosal irritation were documented.

40% of the patients had irregular findings in sniffin’-sticks test. In 8% of the patients, a severe nasal obstruction was detected by active anterior rhinomanometry. In more than 50% of the patients, the evaluation of MRI scans showed swellings of the mucosa of the maxillary sinus and anterior ethmoid cells of more than 3 mm, which was interpreted as a sign of acute sinusitis. The modified Lund-Mackay score was 9 on the average, which is well above the score expected for healthy normal people.

The gold standard of diagnosis is the histological evidence of EGPA-specific changes. In nasal biopsies, no specific histological changes can be detected (so far) except eosinophilia. However, the diagnosis of vasculitis in patients with a history of allergic rhinitis and chronic rhinosinusitis with polyposis and so-called late-onset asthma should make think of EGPA.

The systemic immuno-modulatory therapy of EGPA primarily consists of administering cortisone optionally in combination with cyclophosphamide and methotrexate or other immune modulators [68], [83], [84], [85], [86], [87], [88], [89], [90].

### 2.7 Other

#### 2.7.1 Sarcoidosis 

**Figure 5:**
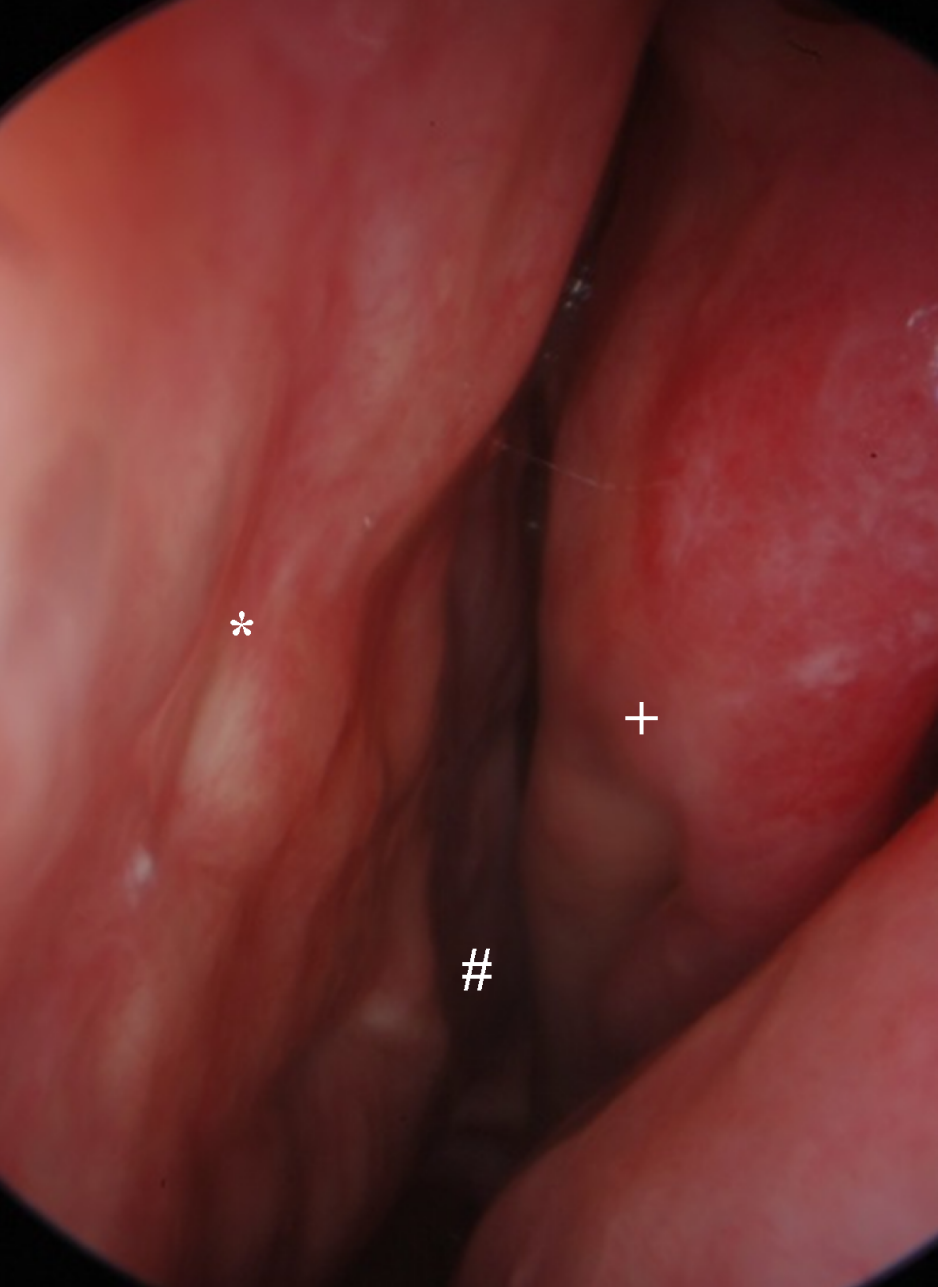
Involvement of the nasal mucosa in sarcoidosis, left nasal cavity (choanal #, nasal septum*, medial nasal concha+)

Sarcoidosis (Figure 5 (Fig. 5)) is a systemic disease of unknown etiology. Currently, an exogenous trigger in genetically susceptible patients (association for haplotype HLA-DRB1+1101) is suspected to be the cause leading to a chronic immune response that leads to the formation of granulomas in the affected organs. Several mediators released by activated macrophages and T lymphocytes contribute to this granuloma formation. Sensitive molecular methods could not detect *Mycobacterium tuberculosis* and *Propionibacterium acnes* in the granulomas although similarities in the disease patterns could suggest this phenomenon. Disorders of vitamin D3 metabolism, hypercalciemia (up to 10% of the patients) and hypercalciuria (up to 35% of the patients) are possible. The preferred age of this disease is the second to fourth decade of life, women are more likely affected than men, and dark-skinned people are affected more frequently and more severely than light-skinned people.

The lungs and the lymphatic system are the preferred sites of manifestation. In half of the cases, there is an extrathoracic manifestation. In these patients, an intrathoracic manifestation can be detected in up to 90% of the cases.

The head and neck region is affected in 10 to 15% of all patients. In the so-called lupus pernio, which must not be confused with the non-specific erythema nodosum, the outer skin of the nose, cheeks, lips, and/or ears is primarily affected. Women are affected more often than men. The plaques are reddish and well perfused. Histologically, granulomas can be detected. Destruction of the underlying supporting structures (bone, cartilage) may occur. A systemic manifestation is possible but not mandatory.

A sinonasal manifestation (1–4% of the patients) often shows a chronic, persistent course. Clinical signs consist of crusts (up to 90% of the patients), nasal obstruction (80–90% of the patients), anosmia (70% of the patients), and epistaxis (about 20% of the patients). Hypertrophy and reddish/purple discoloration with granulations of the septum and inferior turbinates are detected in about half of the patients, nasal polyps are observed in 40%, synechia, septal perforation, and external nasal deformities in approximately 10% of the patients. Epiphora, post-nasal drip, and cephalgia join in as non-specific symptoms. Oro-nasal fistula may develop. By means of CT or MRI scan, sinonasal changes can be identified in up to 85% of the patients (nodular lesions, shading, mucosal swelling, destruction).

The diagnosis of sarcoidosis is made clinically/radiologically and by histological evidence of non-caseating granulomas. A CD4/CD8 T lymphocyte ratio of more than 3.5 in the broncho-alveolar lavage fluid, the detection of soluble angiotensin converting enzyme of two times more than the standard or changes in calcium metabolism can support the diagnosis.

Kveim-Siltzbach’s test is sensitive but only rarely performed. In this context, tissue affected by sarcoidosis is applied intradermally and in sarcoidosis patients granulomatous lesions corresponding to sarcoidosis can be identified histologically within a few weeks in contrast to healthy controls.

The course of the disease is highly variable with most of the patients (30–70%) experiencing a spontaneous remission. In severe cases, immuno-modulatory therapy with corticosteroids or in refractory cases with cytotoxic agents (methotrexate, Azathioprin, cyclophosphamide, and chlorambucil), anti-malarial drugs (chloroquine), thalidomide, or TNF-alpha inhibitors (infliximab, adalimumab, etanercept) is performed.

Local treatment of sinusitis/sinonasal manifestation might be done by (intralesional) corticosteroid application. In refractory cases with obstructive symptoms, surgical techniques might be necessary for treatment. Supportive therapy with crust-dissolving agents may be helpful (nasal ointment, NaCl rinsing). Reconstructive surgery should be performed in remission if indicated [11], [91], [92], [93], [94], [95].

#### 2.7.2 Rosacea

**Figure 6:**
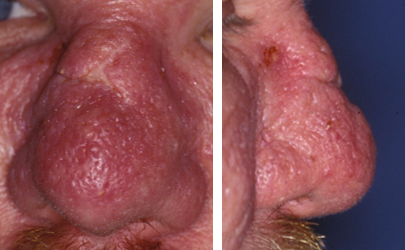
Rhinophyma in rosacea

Rhinophyma (Figure 6 (Fig. 6)), the involvement of the nasal skin in rosacea is a rare manifestation of the disease frequently observed in Europe (prevalence of 1–10%). Between the ages of 30 and 50, women are generally most affected by the disease, but rhinophyma is over-represented in men over 40 years. 

The etiology of the disease is unknown. Predisposing factors are:

- Genetic factors (positive family history in 30%)
- Food (chocolate, nuts, alcohol, cheese)
- Medication (amiodarone, topical steroids)
- Environmental factors (sun, humidity, wind)
- Hormone status (pregnancy, menstruation)

These factors trigger the innate immune system, leading to changes in vascular structures and the release of reactive oxygen species. Although alcohol can be a trigger for the individual, the historical idea of the so-called “drunkard nose” must be statistically refuted.

Current studies demonstrate an incomplete picture of a complex disorder of the inflammatory response of the skin to various stimuli in genetically susceptible patients. An altered dermal vascular system with expansion and dilation of the blood and lymphatic vessels with increased expression of vascular endothelial growth factor (VEGF), the VGF receptor and lymphatic endothelial markers D2-40 seem to be the cause of the telangiectasia and erythema in the affected areas. Furthermore, increased concentrations of the local inflammatory response modulating cathelicidin (enhancing the angiogenetic activity, supporting leukocytes, enhancing vascular permeability) could be detected in the skin. The role of reactive oxygen species is still unclear. Treatment success with antioxidants seems to emphasize the importance of the detected redox status changes in the affected skin. The pathophysiological significance of the detection of mites and the association to *Bacillus oleronius* seems to have a meaning in terms of a trigger for immune response. Clinical studies on the importance of *Helicobacter pylori* are confusing. The importance of the neuro-immune interaction is subject of current research. In the development of rhinophyma fibroblasts, mast cells, T cells, macrophages, and even keratinocytes seem to be actively involved.

The phases of the disease can be divided into a prodromal stage and three further stages. In stage 1, erythema and telangiectasia on the face can be detected, lasting for hours to days. Patients often report about stabbing pain. In stage 2, persistent erythema, telangiectasia, papules, and pustules are documented. In stage 3, inflammatory nodes and hypertrophy of the connective tissue are evident. This hypertrophy of the connective tissue, associated with hypertrophy of the sebaceous glands, leads to the image of rhinophyma predominantly involving the distal two thirds of the nose. This is the most common site of a phyma, others are the forehead, chin, ears, and cheeks. Some authors refer to these findings as fourth stage of rosacea. Evidence regarding the coincidence of malignant tumors is not uniform.

Avoiding triggering factors is the crucial individual goal of therapy. The stages of the disease require a coordinated treatment of local over systemic chemotherapy (special sun blockers, antibiotics, benzoyl peroxide, azelaic acid), laser and light therapy to surgical therapy. The incipient rhinophyma may respond to oral therapy with antibiotics or isotretionin (13-cis-retinoic acid). Advanced stages require surgical therapy (scalpel, electrocautery, cryosurgery, dermabrasion, laser surgery, and others) [96], [97], [98], [99], [100], [101], [102], [103].

#### 2.7.3 Cocaine-induced midline destruction (CIMD)

The 12-month prevalence of cocaine abuse in Germany is 1%. In Europe, about 13 million people have used cocaine at least once in their life. The most common application is transnasally snorted powdered cocaine leading to crusting of the nasal mucosa. The consequence is mechanical cleaning and thus micro-trauma to the mucosa as well as ischemic and direct traumatic effects of cocaine crystals and additives. However, destruction of cartilage and bone of the nose and paranasal sinuses, palate, and surrounding structures is rare. Bacterial infection occurs frequently. The so-called cocaine-induced midline destruction (CIMD) is clinically indistinguishable from other destructive processes. Remarkably in almost all CIMD patients, in contrast to cocaine users without CIMD, ANCA could be detected with specificity against human neutrophil elastase (HNE) and only 5% of all cocaine users develop destruction of the midface.

Perhaps the development of ANCA is causally involved in the development of CIMD and caused by the prolonged bacterial colonization. The effect of HNE-ANCA does not seem to be due to the possible influence on HNE metabolism. Rather HNE-ANCA leads to an increased inflammatory response to injury. Furthermore, cocaine leads (depending on the dose and time) to an apoptosis of mucosal epithelial and inflammatory cells, and this effect may be amplified in CIMD patients. The non-degradation of these inflammatory apoptotic cells is disrupted, however, with the influence of HNE-ANCA and increases the local inflammation and necrosis.

Patients complain about nasal obstruction, epistaxis, facial pain, necrotizing, ulcerative endonasal lesions, crusts, destruction, and hyposmia. In severe defects, dysphagia, nasal reflux as well as ocular and cerebral affection are observed.

Histologically, CIMD is hard to distinguish from GPA. Only exclusion/detection of granulomas in the connective tissue, giant cells, micro-abscesses, and deep necrosis may lead to the correct diagnosis.

The autoimmune reactions (formation of ANCA and anti-phospholipid antibody formation to levamisole, an additive commonly used) in subgroups of cocaine users cannot be influenced sufficiently by immuno-modulatory therapies. Local measures (careful cleaning, NaCl rinsing, soft ointments) relieve the local discomfort. Only the elimination of the harmful substances and the control of local infection, however, can stop the destructive process. Epithesis can alleviate functional disorders. Most authors claim a cocaine abstinence (biochemically controlled if necessary) of 6–12 month before surgical reconstructive measures are performed [104], [105], [106], [107], [108], [109], [110], [111].

#### 2.7.4 Nasal extranodal NK/T cell lymphoma 

**Figure 7:**
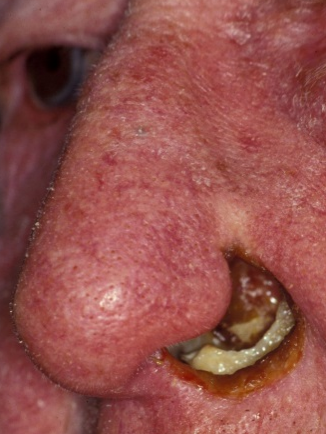
Nasal extranodal natural killer cell/T cell lymphoma with destruction of the midline

Nasal extranodal natural killer cell (NK)/T cell lymphoma (Figure 7 (Fig. 7)) is a centrally situated Non-Hodgkin lymphoma. It is rare in Europe, but the most common malignant lymphoma in the sinonasal field. In Asian and Central and South American populations the incidence is higher than in Europe. The global distribution is parallel to the incidence of EBV infection. The preferred age is the 5^th^ decade. Men are three times more frequently affected than females. NK/T cell lymphomas are characterized by an angiocentric, angiodestructive necrosis. If left untreated, destruction of the upper aerodigestive tract, the ocular system, and cerebral involvement develop rapidly. Systemic progression involving the skin, soft tissue, lungs, gastrointestinal tract, and testes occur and have a worse prognosis than the localized form. Nasal extranodal NK/T cell lymphoma has a strong association with EBV infection. The pathogenic role of the virus detection is controversial to date (coding of substances which have a homology to and interaction with anti-apoptotic molecules, cytokines, and neurotransmitters and act as immortalization of the infected cells). The prognostic significance (a high titer correlated with the spread of the disease, an intractable course, and poorer survival) is widely accepted. Chromosomal changes in chromosome 6 seem to be responsible for the disease-specific FOXO3 anomalies that support the lymphoma genesis. In the prodromal stage, patients complain of nasal obstruction, serous rhinorrhea, and swelling over the nose and cheeks. Nonspecific radiographic changes can be detected at this time. In the active stage, which may extend over months or even a few years, ulcerative changes of the infiltrates of the skin and mucous membranes appear, and infiltration of adjacent tissue is common. The ulcers develop into destruction of the midface with participation of sensitive adjacent structures. Arrosion of the ethmoid arteries and the terminal branches of the maxillary arteries can lead to severe bleeding. The lesions are often painless.

The diagnosis is primarily secured by histological examination. In this context, angiocentric, angiodestructive tissue necrosis can be detected in addition to superficial necrosis. The image of the neoplastic lymphoid cells (CD56/CD3 positive) and cytotoxic T cells is various with high proliferation rate. Cytotoxic molecules such as granzyme B and perforin TiA1 can be displayed regularly. EBV-DNA can be mostly revealed. To exclude other T cell lymphomas, the detection of clonal rearrangements is useful. An accompanying inflammatory response may complicate the histological diagnosis. Imaging techniques can show the local and systemic spread. A bone marrow punch to exclude dissemination of the disease should be performed.

The prognosis with a median survival of 7.8 months is worse compared to other T cell lymphomas. The 5-year survival rate amounts to 40%. Remissions can be achieved by hyperfractionated radiotherapy but also by administering chemotherapy like CHOP (cyclophosphamide, vincristine, doxorubicin, and prednisolone). The success of this therapy depends on the plasma load of EBV-DNA, B-symptoms, local lymphadenopathy, the International Prognostic Index (IPI), and the expression of the tumor cells of P-glycoprotein (which exports various chemotherapeutic agents actively out of the cells). The usefulness of the treatment options [radio-chemotherapy, IFNa-plus ultraviolet B phototherapy, stem cell transplantation, and SMILE (steroid methotrexate, ifosfamide, L-asparaginas, and etoposide)] is currently being evaluated. Radio-chemotherapy with anthracycline or L-asparaginase is recommended [112], [113], [114], [115], [116], [117].

#### 2.7.5 Cholesterol granulomatosis

Worldwide, 135 cases of cholesterol granuloma of the sinonasal system are described. More often cholesterol granulomas are detected at the lateral skull base but also in internal organs. Histologically, the granuloma is characterized by a chronic granulomatous tissue reaction to cholesterol crystals. In contrast to cholesteatoma, no epithelial structures can be demonstrated. Plasma cells, giant cells, histiocytes, and lymphocytes are detected regularly and the presentation of haemosiderin and other blood breakdown products speaks for previous history of hemorrhage. Limitations of ventilation and drainage disabilities are crucial etiological factors. However, the presence in internal organs allows expecting further/other crucial factors.

The median age is 44 years, men are 6 times more frequently affected than women. The symptoms are: orbital pain (66%; pain, visual loss, axis deviation, exophthalmos), cephalgia (19%), nasal obstruction (17%), rhinorrhea (9%), and epistaxis (4%). Only few patients (4%) are asymptomatic. Evidence of clear-yellowish rhinorrhea with detection of cholesterol crystals may represent a specific sign for the presence of a cholesterol granuloma. A history of previous surgery or trauma can be found in only 10–15% of the patients. However, often chronically rhinosinusitic complaints or mucociliary dysfunction in the history are described. The cholesterol granulomas manifest in the frontal sinus (60%), maxillary sinus (34%), ethmoid sinus (4%), and sphenoid sinus (2%). CT scan proves bone arrosion in 80% of the cases as well as shading and mucosal swelling. In MRI, the lesions show an intense signal in the T1 and T2 weighted series (possibly caused by the accumulation of methemoglobin).

Therapy consists of microsurgical removal and restoring of a sufficient ventilation. In the literature, osteoplastic approaches (80%) are more frequently described than endonasal approaches (20%). This may be due to the favored access routes of different medical subspecialties. Low recurrence rates are described for either approach.

Postoperatively, more than 90% of the patients are symptom-free (follow-up of 35 months) [118], [119], [120], [121].

## 3 Disturbed mucociliary clearance

The composition of the mucus as well as the function of the cilia is crucially involved in the undisturbed mucociliary clearance of the airways. The physical barrier may be disturbed temporarily or permanently at different levels. Temporary functional limitations and/or permanent functional deficits may be the consequence with possible destruction and sometimes fatal outcome (Table 2 (Tab. 2)).

### 3.1 Primary ciliary dyskinesia

Several predominantly autosomal recessive inherited diseases with persisting functional and/or morphological changes of the cilia are subsumed under the diagnosis of primary ciliary dyskinesia (PCD). Disease of the non-motile cilia are not discussed in this context. Currently more than 20 genes have been described whose defect leads to PCD. Precise epidemiological data are not available. Prevalence estimates vary between 1/2,000 and 1/40,000. Ciliary structures are found in humans in the mucous membranes of the respiratory tract, the ependymal cells of the central nervous system, the fallopian tubes, the sperm, and the embryonic node. Ultrastructurally, the cilia show 9 peripheral and 2 central double tubules connected by outer and inner dynein arms and radial spokes as well as other connections. This is the morphological prerequisite for unhindered function. The cilia of the embryonic node show a 9+0 configuration. The healthy beat frequency of the cilia is 6–12 Hz.

In addition to the lungs, which is leading organ regarding complaints, impairment of the middle ear, nose and paranasal sinuses, genitourinary tract, central nervous system, and right-left orientation are detectable. Disorders of the ciliary function in the sinonasal system manifest clinically in neonatal rhinitis and nasal obstruction (about 50%), mucopurulent rhinitis, chronic (polypoid) sinusitis (up to 100%), and dysosmia (which could also be caused by synchronous disturbances of the olfactory receptor organs). Morphologically, the sinuses often are dysplastic or aplastic.

In addition to the critical evaluation of the clinical symptoms, which might be recognized early and a possible positive family history, the following investigations are helpful to confirm the diagnosis:

- Nasal nitric oxide (NO) measurements show a very low concentration, however, decreased NO concentrations are also measured in inflammatory sinus disease and cystic fibrosis. The lack of standardization of the examination and the necessary active cooperation of the patient are (so far) limiting factors of the examination.
- Using brush or scratch biopsies of the mucosa of the inferior turbinates, the ciliary beat frequency can be determined by microscopic analysis subjectively and objectively, and the movement patterns are analyzed. These studies are prone to failure caused by acute inflammatory processes or exogenous noxious agents and other systemic influences (vitamin A deficiency, sulfur dioxide, formaldehyde etc.). By repeated examinations or investigations of primary cell cultures, these confounding factors can be minimized.
- By means of transmission electron microcopy (TEM), the ultrastructure of the cilia can be studied. However, recent data suggest that the ultrastructure in the electron microscopic examination appears regular in 30% of the patients although genetic defects are present that encode cilia components.
- Currently, antibodies against various cilia target structures are to allow immuno-fluorescence based studies.
- Genetic analyses of PCD patients show heterogeneous results. Between 1999 and 2011, 12 genes have been described with disease association. New methods of genetic analysis in particular next generation sequencing led to the description of 15 new genes since 2012. Most of the changes are so-called “loss-of-function” mutations that are usually only individually detectable. Very few mutations can be detected in independent patients. Some of the changes show genotype-phenotype correlations. Evidence of genetic mutations that lead to the disorder of the central tubules cause a disruption of the ciliary function, but no right-left orientation defects (9+0 structure of the cilia of the embryonic node). Today the genetic analyses lead to quick results and in the future they could facilitate the diagnosis and shorten the diagnostic process (especially in patients with incomplete clinical appearance).

Besides, the primary diagnosis, the periodic evaluation of the patient is crucial in order to adjust the therapy and to positively influence the progression, in particular the lung involvement. However, the proposed therapeutic interventions are not evidence-based.

Chronic rhinosinusitis should also be evaluated regularly and therapy should be adapted. Nasal steroids, irrigation with NaCl solutions and the bacterial spectrum matched systemic and possibly local antibiotic treatments should be applied if necessary. Avoiding ciliary function disturbing exogenous stimuli (tobacco smoke, low humidity etc.) is critical. Surgical techniques (e.g. with atypical fenestration of the inferior nasal passage) for drainage and ventilation of the sinus are the last resort. With the mentioned therapeutic interventions, solely symptoms of the underlying pathophysiological processes are influenced. In the (distant) future, with the results of the genetic analyses, a personalized gene/stem cell therapy may lead to a curative treatment of the disease. However, this aspect represents a major challenge, especially for PCD with its various heterogeneous genetic changes [122], [123], [124], [125], [126], [127], [128], [129], [130], [131].

### 3.2 Cystic fibrosis (CF)

Mutations of the cystic fibrosis transmembrane conductance regulator (CFTR) gene cause the autosomal recessive inherited disease of cystic fibrosis. So far, about 1939 mutations have been described for this multi-organ disease (affecting lung, pancreas, liver/gall bladder, vas deferens etc.). The median life expectancy is 40 years. So-called class I–III mutations correlate with a severe form of cystic fibrosis, class IV–V with a milder phenotype. Mutations of the CFTR gene in heterozygote carriers lead to an increased rate of chronic sinonasal diseases (about 30% of the examined persons). Patients with class I–III mutations have a significantly smaller frontal sinus and sphenoid sinus, more shading of the sinuses and often an osteitis/neoosteogenesis of the wall of the maxillary sinus compared to patients with class IV–V mutations.

Delta F508 homozygous CF patients show frequent involvement of the sinonasal system with a greater likelihood (relative risk of 2.33) for sinus surgery and frequently demonstrate dysplasia of the paranasal sinuses.

CFTR mutations have an impact far beyond a disturbance of AMP-regulated chloride channels of epithelial cells and can for example lead to inflammatory response, independent of infections and NF-kappa B mediated.

The diagnosis is made on the evaluation of clinical symptoms, the so-called sweat test (chloride content ≥60 mmol/l), molecular genetic and electrophysiological examinations (measurement of the nasal transepithelial potential difference or intestinal short circuit current measurement) in the order listed. The nasal N0 measurement is not of importance in the current S2 guideline entitled “Diagnosis of cystic fibrosis”.

Following the concept of the common or universal airway, changes the upper respiratory tract also affect the lower respiratory tract, although this has not been proven for cystic fibrosis in particular.

63% of adult CF patients suffer from rhinosinusitis.

The sinonasal symptoms described are non-specific: nasal obstruction (80%), rhinorrhea (more than 50%), cephalgia (50%), dysosmia (about 25%), cough, and halitosis. Signs detected by endoscopy are hyperplasia of the nasal turbinates (almost 90%), bulging of the lateral nasal wall, a prominent uncinate process, and nasal polyps (7–48%). Notably, the inflammatory response in the polyps of cystic fibrosis patients is predominantly neutrophilic (and not eosinophilic as usually in Europe). This circumstance might be useful for diagnostics by examination of nasal lavage. The risk for the development of polyps appears to increase with age. Amazingly, children who show nasal polyps appear with evidence of a lower pulmonary severity of the disease. In contrast, the colonization rate of the lung with *Pseudomonas aeruginosa* seems to be increased in CF patients with nasal polyps. CT scans frequently reveal protrusions of the lateral nasal wall, demineralization of the uncinate process, and hypo- or aplasia of the paranasal sinuses (frontal sinus agenesis in up to 60%). By means of microbiological investigations in younger patients, usually *Staphylococcus aureus* and *Haemophilus influenzae* and in elderly patients *Pseudomonas aeruginosa* are detected. In 25–44% of the patients with sinonasal complaints, a multimicrobial infection occurs. Increased adhesion rates of the micro-organisms may be due to CFTR mutation caused by changes of receptor proteins of the cell surface. 50–70% of the proven sinonasal germs can be simultaneously identified in the deeper airways. This result supports the theory that a positive effect on the lower respiratory tract can be achieved by influencing the sinonasal system. Therapeutic approaches regarding the treatment of sinonasal involvement in cystic fibrosis have been examined only rarely by randomized controlled trials (RCT). Rinsing with NaCl, topical or systemic decongestants, antihistamines, mucolytical substances such as N-acetyl cysteine, systemic corticosteroids, topical aminoglycosides, anti-inflammatory drugs (ibuprofen), and systemically applied macrolides are used, but often they are not evaluated with sufficient accuracy. The topical application of 100 µg of betamethasone twice daily in a controlled RCT study resulted in a significant reduction of the symptoms and endoscopically controlled size of the polyps in the treated group. A long-term control of this effect, however, was not performed. The application of Dornase alfa after functional endoscopic sinus surgery (FESS) is also investigated in a RCT. After one year, a more pronounced improvement of the nasal symptoms, endoscopic findings, and the assessment of the paranasal sinuses in CT scans in the treated group was documented and compared to the placebo-treated group.

The role of surgical intervention is critically discussed in the current literature. Possible useful indications are:

- Persistent nasal obstruction despite medical intervention
- Anatomically related obstruction of the paranasal sinuses
- Correlation between symptoms of the sinonasal system and pulmonary exacerbations
- Symptoms such as cephalgia and facial pain impairing the quality of life

The incidence of mucoceles in cystic fibrosis patients is reported with up to 4%. The mucoceles can occur early (6^th^ month of life). Surgical therapy usually leads to healing of mucoceles. Surgical treatment of chronic rhinosinusitis with subsequent locally applied tobramycin might support the success of lung transplantation. Further studies will need to clarify whether the local application of gentamycin in addition to the antimicrobial effect corrects the translation of CFTR gene in cystic fibrosis patients with premature stop codons in vivo and affect the (local) disease progression. The first attempt of gene therapy was carried out in the 1990ies. Since then, 20 other studies followed. Various challenges for this causal therapy remain unresolved and are subject of intense research (optimal gene transfer via the (modified) mucosal barrier, definition of the target cell, optimal gene expression rate, definition of meaningful outcome parameters) [132], [133], [134], [135], [136], [137], [138], [139], [140], [141], [142], [143].

## Notes

### Competing interests

The author declares that he has no competing interests.

### Acknowledgements

Sabine Lübker for her assistance in literature acquisition, Prof. Regine Gläser for providing images, Prof. Rainer Podschun for his critical comments on the manuscript, Henrik Andersen for logistic support.

## Figures and Tables

**Table 1:**
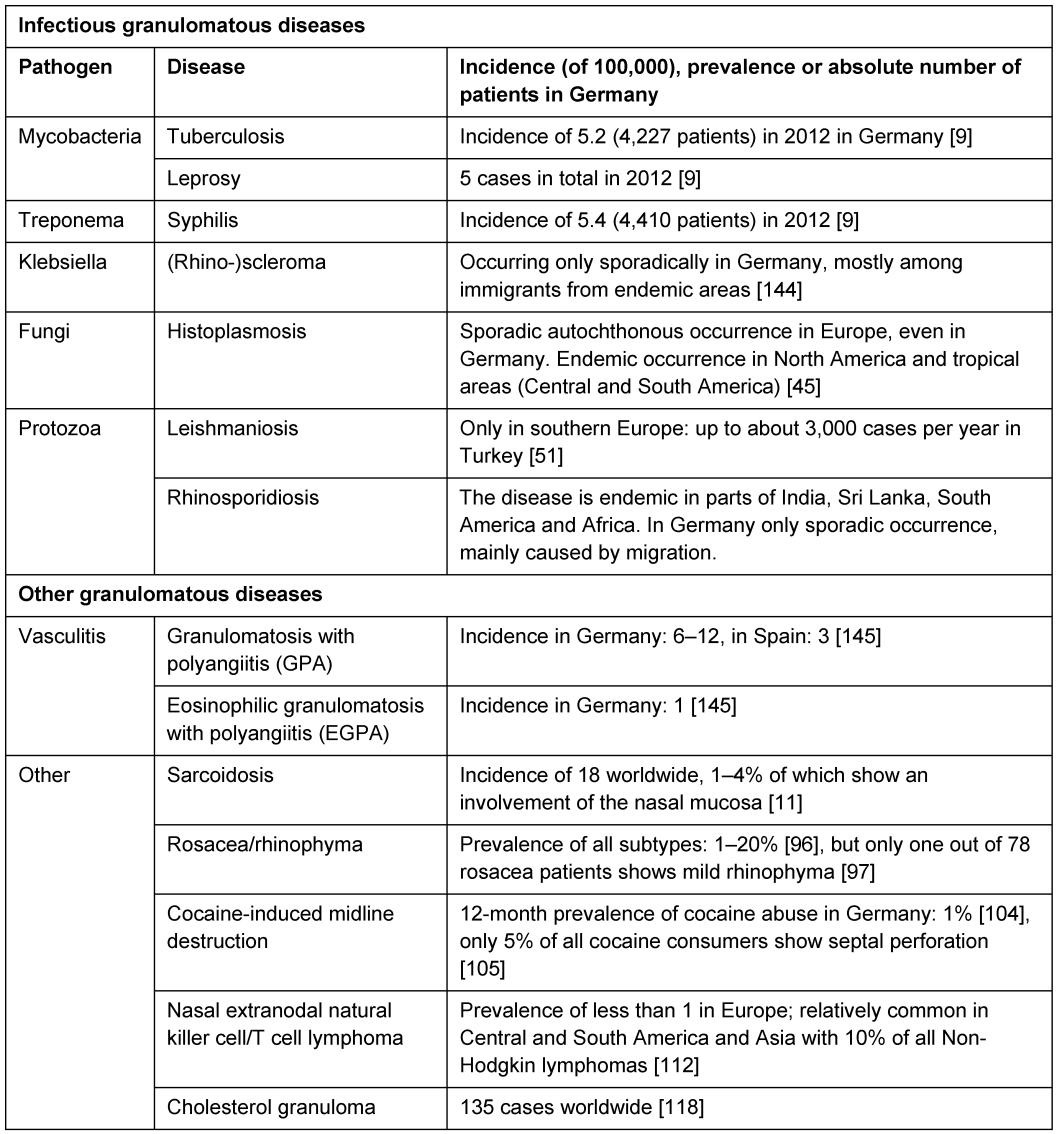
Pathogens, diseases, and incidence, prevalence or number of patients of granulomatous diseases in Germany

**Table 2:**
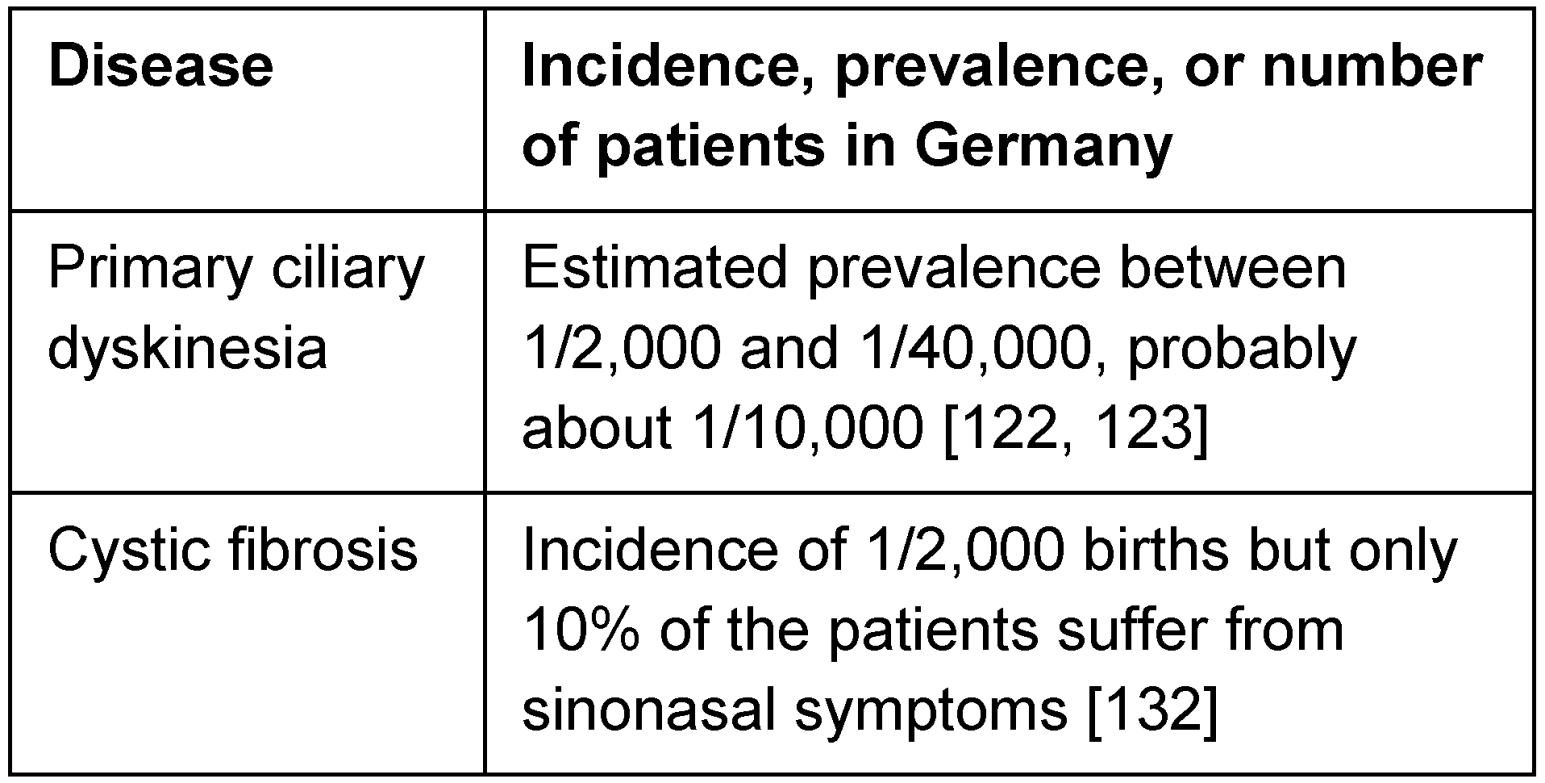
Ciliary dysfunction
